# Effect of berberine on lipopolysaccharide-induced monocyte chemotactic protein-1 and interleukin-8 expression in a human retinal pigment epithelial cell line

**DOI:** 10.1007/s10792-017-0697-x

**Published:** 2017-08-29

**Authors:** Hu-Shan Cui, Yu-Min Li, Wei Fang, Jiu-Ke Li, Yuan-Min Dai, Lian-Shun Zheng

**Affiliations:** 1 Department of Ophthalmology, Affiliated Sir Run Run Shaw Hospital, School of Medicine, Zhejiang University, No. 3 Qingchun Road East, Hangzhou, 310016 Zhejiang Province China; 2 Institute of Anatomy and Cell Biology, School of Medicine, Zhejiang University, Hangzhou, China

**Keywords:** Berberine, Human retinal pigment epithelial cells, Lipopolysaccharide, Monocyte chemotactic protein-1, Interleukin-8

## Abstract

**Purpose:**

In this study, we elucidated the effects of berberine, a major alkaloid component contained in medicinal herbs, such as *Phellodendri Cortex* and *Coptidis Rhizoma*, on expression of monocyte chemotactic protein-1 (MCP-1) and interleukin-8 (IL-8) in a human retinal pigment epithelial cell line (ARPE-19) caused by lipopolysaccharide (LPS) stimulation.

**Methods:**

ARPE-19 cells were cultured to confluence. Berberine and LPS were added to the medium. MCP-1 and IL-8 mRNA were measured by real-time polymerase chain reaction. MCP-1 and IL-8 protein concentrations in the media were measured using enzyme-linked immunosorbent assay.

**Results:**

After stimulation with LPS, MCP-1 and IL-8 mRNA in ARPE-19 cells reached maximum levels at 3 h, and MCP-1 and IL-8 protein in the culture media reached maximum levels at 24 h. Berberine dose-dependently inhibited MCP-1 and IL-8 mRNA expression of the cells and protein levels in the media stimulated with LPS.

**Conclusions:**

These findings indicate that berberine inhibited the expression of MCP-1 and IL-8 induced by LPS.

## Introduction

The retinal pigment epithelial (RPE) cells play important roles in physiology of the retina. The main functions of RPE are phagocytosis of photoreceptor segments, absorption of light, and formation of blood–retina barrier, which regulates the ionic and metabolic gradients required for normal retinal function [1]. The RPE cells also have an important role in different pathologic processes of the retina such as age-related macular degeneration and diabetic retinopathy [2–5]. Human RPE cells isolated from donor eyes express monocyte chemotactic protein-1 (MCP-1) and interleukin-8 (IL-8) by stimulation with lipopolysaccharide (LPS) [6, 7]. MCP-1 is a potent chemoattractant inducing the infiltration of monocytes and macrophages into tissues [8]. IL-8 is known as a neutrophil chemotactic factor [9]. MCP-1 and IL-8 are member of two chemotactic families [10] and are called CCL2 and CXCL8, respectively, according to a new classification system recommended by Zlotnik and Yoshie [11]. Crane et al. [12] reported that MCP-1 and IL-8 are produced at much higher levels than other chemokines tested in RPE cells. Dunn et al. [13] reported that ARPE-19, a human retinal pigment epithelial cell line, has structural and functional properties characteristic of RPE cells in vivo. Our study showed that MCP-1 and IL-8 in the culture media of ARPE-19 cells are increased by IL-1β or TNF-α [14, 15]. LPS induced MCP-1 and IL-8 in ARPE-19 cells [7, 16, 17]. The levels of chemotactic cytokines including MCP-1 and IL-8 are higher in the vitreous of patients with age-related macular degeneration [18–20], proliferative vitreoretinal diseases [21, 22], and proliferative diabetic retinopathy than in normal subjects [23–25]. These cytokines may help in stimulating the infiltration of monocytes and macrophages into eyes with such disorders [26]. Thus, MCP-1 and IL-8 may be involved in part in the pathogenesis of intraocular disorders.

**Fig. 1:**
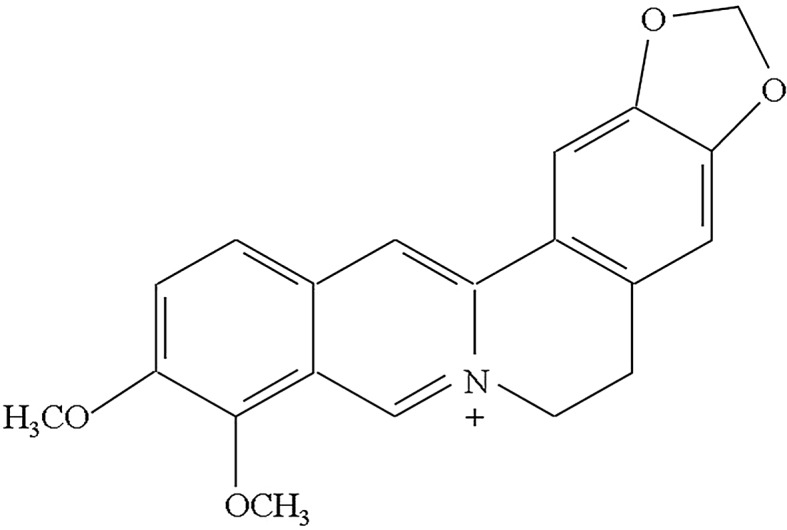
Chemical structure of berberine

Berberine (Fig. 1) is a major alkaloid isolated from medicinal herbs such as *Berberis* sp., *Coptidis* sp. rhizome, and *Phellodendri* sp. cortex; these plants are included in several Kampo formulae (traditional Chinese–Korean–Japanese medicine), such as Oren-gedoku-to (Huang-Lian-Jie-Du-Tang), and have been used to treat various inflammatory diseases. This alkaloid has multiple pharmacological actions, including diarrhea-treating action [27–29], anti-inflammatory effects [15, 30–36], glucose-lowering potential [37–40], cholesterol-lowering effect [41–43], and neuroprotective action [44–47]. We previously reported that berberine inhibited MCP-1 and IL-8 induced by LPS in rat uveitis in vivo [36]. In the present study, we investigated the effects of berberine on MCP-1 and IL-8 expression in ARPE-19 cells stimulated with LPS.

## Materials and methods

### Cell culture

ARPE-19, a human RPE cell line, was purchased from American Type Culture Collection (No. CRL-2302, Rockville, MD, USA). The cells were maintained in Dulbecco’s modified essential medium and Ham’s F12 (DMEM/F12; 1:1; Gibco-BRL, NY, USA) supplemented with 10% fetal bovine serum (FBS); penicillin, 100 U/ml; and streptomycin, 100 μg/ml, to obtain confluent cells. All cells were cultured at 37 °C under 10% CO_2_ and 90% moist air. Media were changed twice a week. The viability of ARPE-19 cells after incubation was assessed using trypan blue dye.

### Total RNA extraction and MCP-1 and IL-8 mRNA expression by real-time polymerase chain reaction

Berberine was purchased from Sigma Chemicals (St. Louis, Mo., USA). The molecular weight of the alkaloid was 371.8, and the chemical structure is shown in Fig. 1. Berberine was dissolved in 50 mM dimethyl sulfoxide (DMSO) just before use. The final concentration of DMSO was kept at ≤0.05% in the culture media to avoid its inhibitory effects on the proliferation of the ARPE-19 cell line.

ARPE-19 cells were planted and cultured in 6-well culture plates (averaging 2.0 × 10^6^ cells/well) for 14 days in DMEM/F_12_ medium containing 10% FBS. After the cells were washed twice with serum-free medium, ARPE-19 cells were preincubated for 2 h in serum-free medium with 0.05% DMSO or 0.2, 1, 5, and 25 μM of berberine for 30 min. Then, LPS (*Escherichia coli*, serotype 055:B5, Sigma-Aldrich, St Louis, Mo., USA) was added to the medium and incubated. Total RNA was extracted from the cells using RNeasy Protect Mini Kit (Qiagen, Hilden, Germany) and treated with RNase-free DNase Set (Qiagen, Hilden, Germany) to remove any residual genomic DNA. One microgram of each total RNA was reverse transcribed using random hexamers, MultiScribe reverse transcriptase (Applied Biosystems, Branchburg, NJ, USA), and thermal cycler (Gene Amp PCR System 2400; Applied Biosystems, Norwalk, CT, USA). Condition of the reverse transcription included incubation at 25 °C for 10 min, reverse transcription at 48 °C for 30 min, and reverse transcriptase inactivation at 95 °C for 5 min.

cDNA was used to detect real-time PCR products for MCP-1 and IL-8 using TaqMan Universal Master Mix (Applied Biosystems, Branchburg, NJ, USA) and ABI PRISM™ 7700 Sequence Detector (Applied Biosystems, Foster, CA, USA) with TaqMan^®^ Pre-Developed Assay Reagents (Applied Biosystems, Foster, CA, USA) for human MCP-1 and IL-8. The thermal profile for each primer consisted of 2 min at 50 °C and 10 min at 95 °C, followed by 40 cycles of 15 s at 95 °C and 1 min at 60 °C. To compare expression patterns, mRNA template concentrations for glyceraldehyde-3-phosphate dehydrogenase (GAPDH) and the target genes were calculated using the standard curve method. The expression levels of MCP-1 and IL-8 mRNA were normalized by GAPDH mRNA level in each sample, and the changes were expressed as an *n*-fold relative to the value of cells untreated with LPS.

### MCP-1 and IL-8 protein concentrations by enzyme-linked immunosorbent assay (ELISA)

ARPE-19 cells were seeded in 24-well culture plates (averaging 2.0 × 10^5^ cells/well) and incubated for 14 days in DMEM/F_12_ medium containing 10% FBS. The cells were washed twice with serum-free medium and incubated in serum-free medium for 2 h. ARPE-19 cells were preincubated in serum-free medium with 0.05% DMSO or 0.2, 1, 5, and 25 μM of berberine for 30 min. Then, LPS was added to the medium, and it was incubated for 24 h. Thereafter, the supernatant was collected and stored at −70 °C until assay. MCP-1 and IL-8 protein concentrations were determined using ELISA (Amersham Biosciences, Little Chalfont, Buckinghamshire, UK) and were calculated based on standard curves using concentrations of recombinant MCP-1 and IL-8 in the ranges of 51–2000 and 25–1000 pg/ml, respectively. All assays were performed in duplicate.

### Statistical analysis

The results were expressed as mean values ± standard errors. Statistical analysis was performed using the Scheffe’s procedure for multiple comparisons of mean value. *P* < 0.05 was considered statistically significant.

## Results

### Expression of MCP-1 mRNA and IL-8 mRNA after stimulation with LPS in ARPE-19 cells

Cell viability was above 98%; most ARPE-19 cells incubated with LPS (5 μg/ml), 0.05% DMSO, and berberine (25 μM) for 24 h were viable.

**Fig. 2:**
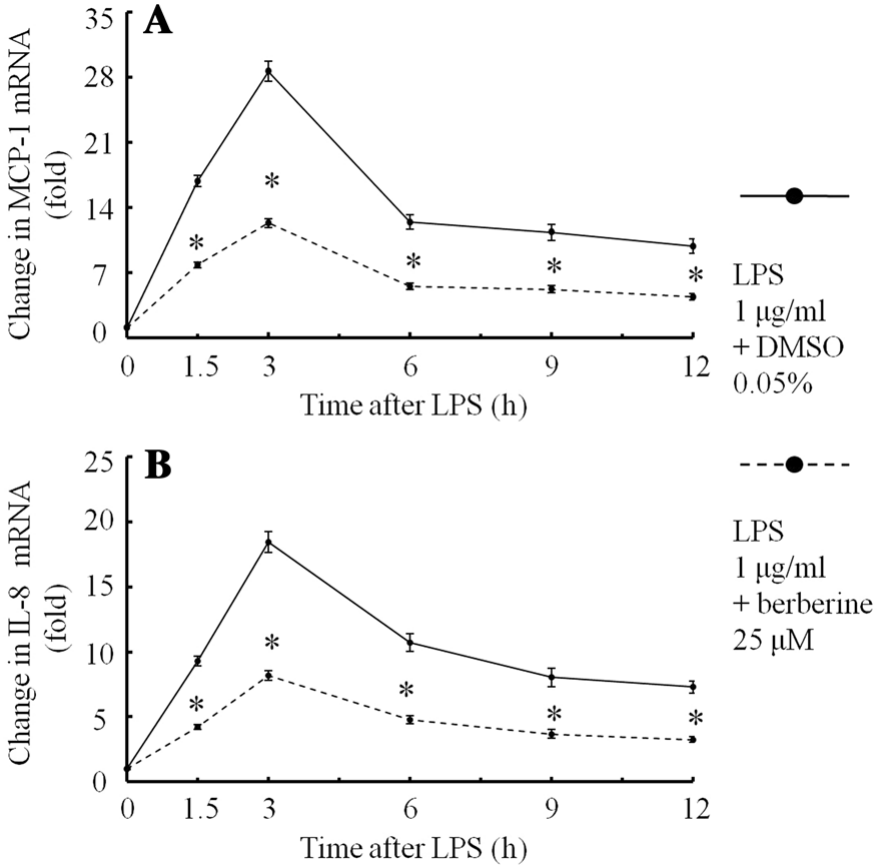
Changes in MCP-1 mRNA and IL-8 mRNA expression after stimulation with LPS in ARPE-19 cells in serum-free media were preincubated for 30 min with berberine (25 μM) or 0.05% DMSO. After LPS (1 μg/ml) had been added to the medium, ARPE-19 cells were incubated for 1.5, 3, 6, 9, and 12 h. The changes in mRNA were determined using real-time PCR. Changes in MCP-1 mRNA (**a**) and IL-8 mRNA (**b**) after stimulation with LPS are shown. The data are expressed as means ± standard errors of four independent experiments. *P < 0.01, compared to the value without berberine

When the medium was incubated with 0.05% DMSO, ARPE-19 cells expressed small amounts of MCP-1 mRNA and IL-8 mRNA. After stimulation with LPS (1 μg/ml) and 0.05% DMSO, MCP-1 mRNA (Fig. 2a) and IL-8 mRNA (Fig. 2b) in ARPE-19 cells increased, reached maximum levels (28.4- and 18.3-fold, respectively) at 3 h, and then gradually decreased. Berberine (25 μM) inhibited these mRNA expressions in ARPE-19 cells (Fig. 2a, b).

**Fig. 3:**
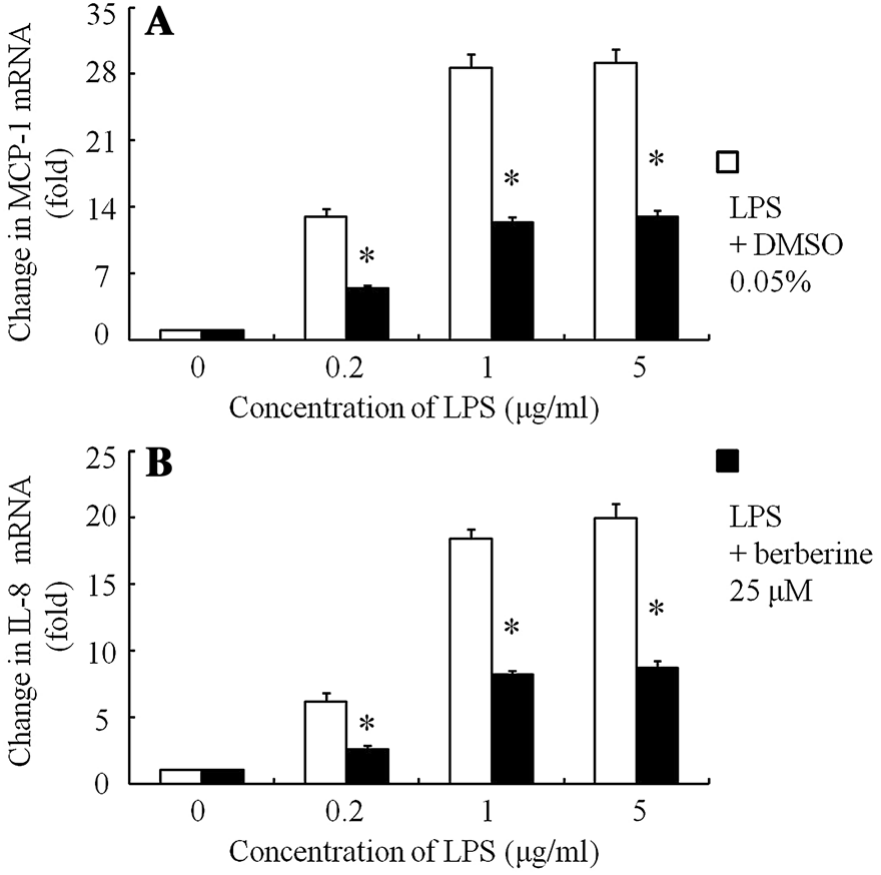
Changes in MCP-1 mRNA and IL-8 mRNA expression after stimulation with various concentrations of LPS in ARPE-19 cells in serum-free media were preincubated for 30 min with berberine (25 μM) or 0.05% DMSO. After various concentrations of LPS (0.5, 1 and 5 μg/ml) had been added to the medium, the cells were incubated for 3 h. The changes in mRNA were determined using real-time PCR. Changes in MCP-1 mRNA (**a**) and IL-8 mRNA (**b**) after stimulation with LPS are shown. The data are expressed as means ± standard errors of four independent experiments. *P *<* 0.01, compared to the value without berberine

LPS (0.2–5 μg/ml) dose-dependently stimulated MCP-1 mRNA (Fig. 3a) and IL-8 mRNA (Fig. 3b) in ARPE-19 cells. Berberine (25 μM) inhibited these mRNA expressions in ARPE-19 cells (Fig. 3a, b).

**Fig. 4:**
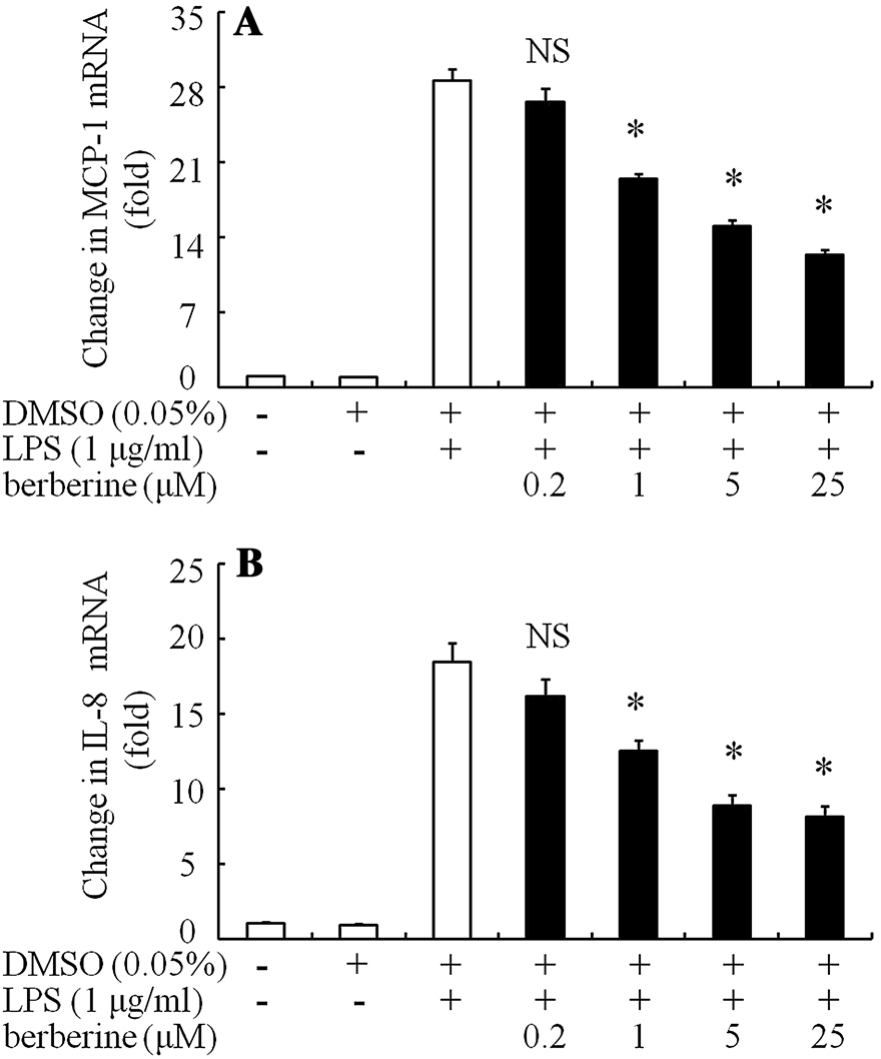
Effects of berberine on MCP-1 mRNA and IL-8 mRNA expression after stimulation with LPS in ARPE-19 cells in serum-free media were preincubated for 30 min with berberine or 0.05% DMSO. LPS (1 μg/ml) was added to the media and incubated for 3 h. After incubation, total RNA was extracted from the ARPE-19 cells, and semiquantitative real-time PCR was performed. Changes in MCP-1 mRNA (**a**) and IL-8 mRNA (**b**) in ARPE-19 cells after stimulation with LPS for 3 h are shown. The data are expressed as means ± standard errors of four independent experiments. *P < 0.01; *NS* not significant, compared to the value of LPS-alone-treated group

Incubation with DMSO 0.05% had only a little inhibition on MCP-1 (Fig. 4a) and IL-8 mRNA (Fig. 4b). Berberine (1–25 μM) dose-dependently inhibited MCP-1 mRNA (Fig. 4a) and IL-8 mRNA (Fig. 4b) in the cells stimulated with LPS (1 μg/ml) at 3 h. Berberine at 25 μM showed maximum inhibition: MCP-1 and IL-8 mRNA expression, 12.2- and 8.2-fold, respectively.

### MCP-1 and IL-8 protein concentrations after stimulation with LPS in ARPE-19 cells

**Fig. 5:**
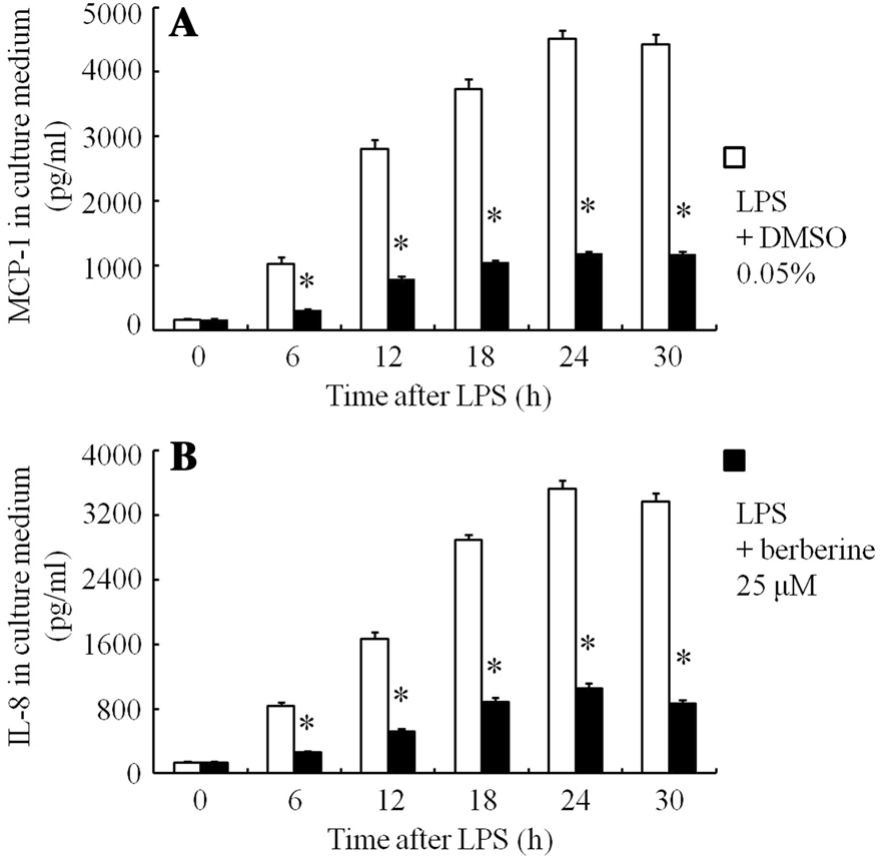
Changes in MCP-1 and IL-8 protein concentrations in culture medium of ARPE-19 cells in serum-free media were preincubated for 30 min with berberine or 0.05% DMSO. After LPS (1 μg/ml) had been added to the medium, the cells were incubated for 6, 12, 18, 24, and 30 h. The MCP-1 levels (**a**) and IL-8 concentrations (**b**) in the culture media after stimulation with LPS, determined using ELISA, are shown. The data are expressed as means ± standard errors of four independent experiments. *P *<* 0.01, compared to the value without berberine

When the cells were incubated for 24 h with 0.05% DMSO, MCP-1 and IL-8 protein concentrations in the culture media were 147 and 131 pg/ml, respectively. After stimulation with LPS (1 μg/ml) and 0.05% DMSO, MCP-1 (Fig. 5a) and IL-8 (Fig. 5b) in the culture media increased and reached maximum levels (4514 and 3529 pg/ml, respectively) at 24 h, and remained so at 30 h. Berberine (25 μM) inhibited these protein levels (Fig. 5a, b).

**Fig. 6:**
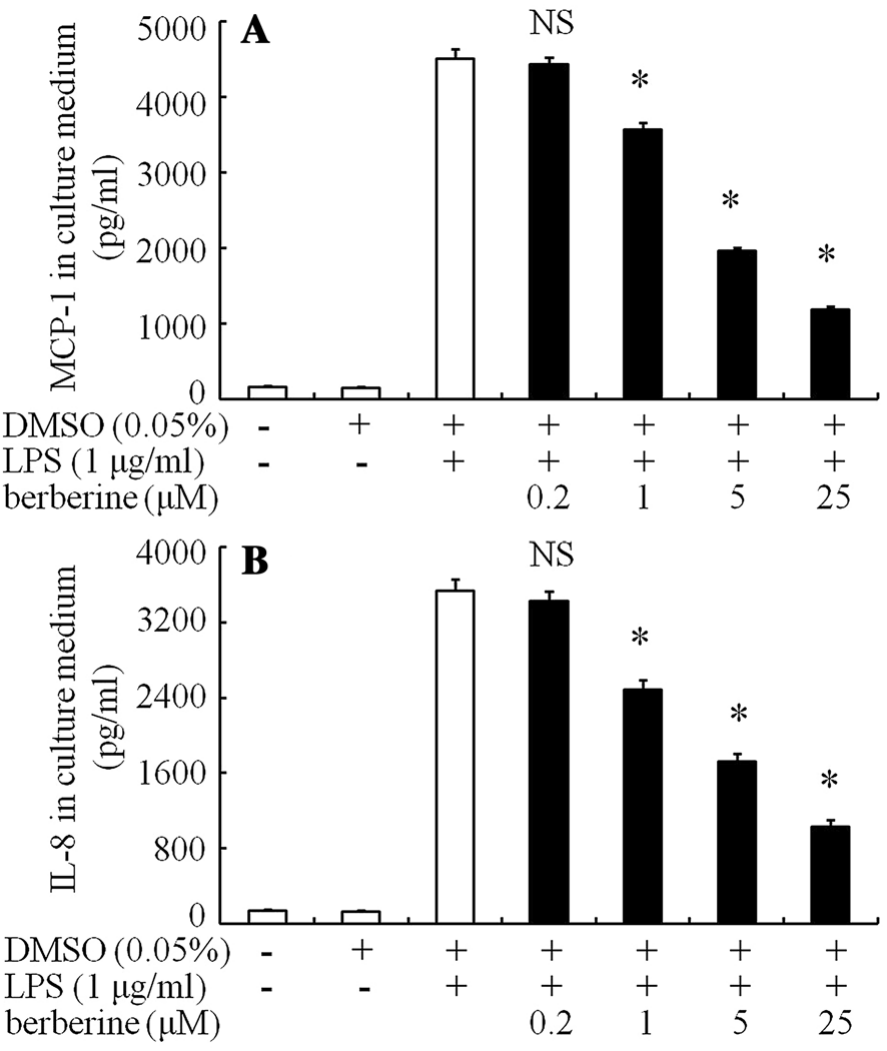
Effects of berberine on MCP-1 and IL-8 protein concentrations after stimulation with LPS in ARPE-19 cells were incubated for 30 min in serum-free media with berberine or 0.05% DMSO. LPS (1 μg/ml) was added to the medium and the cells were incubated for 24 h. The MCP-1 levels (**a**) and IL-8 protein concentrations (**b**) after stimulation with LPS in the culture media, determined using ELISA, are shown. The data are expressed as means ± standard errors of four independent experiments. *P < 0.01; *NS* not significant, compared to the value of LPS-alone-treated group

DMSO 0.05% had only a little inhibition on IL-8 and MCP-1 protein levels in the media (Fig. 6). Berberine (1–25 μM) dose-dependently inhibited LPS-stimulated MCP-1 (Fig. 6a) and IL-8 (Fig. 6b) concentrations in the culture medium at 24 h. Berberine at 25 μM showed maximum inhibition: MCP-1 and IL-8 protein levels, 1182 pg/ml (inhibition of 73.8%) and 1034 pg/ml (inhibition of 70.7%), respectively.

## Discussion

Dunn et al. [13] have demonstrated changes in gene expression and function with time of confluence in ARPE-19 cells with a mature epithelial phenotype require as much as 2–4 weeks of culture. In the present study, therefore, ARPE-19 cells cultured for 14 days after planting were used.

In the our present findings, 0.2, 1, and 5 μg/ml LPS dose-dependently stimulated MCP-1 mRNA and IL-8 mRNA, and 5 μg/ml LPS showed had no significant difference compared with 1 μg/ml LPS in ARPE-19 cells. Therefore, the inhibitory effects of berberine were determined after stimulation of 1 μg/ml LPS in the present study. In the present study, after stimulation with LPS (1 μg/ml), MCP-1 and IL-8 mRNA in ARPE-19 cells reached peak levels at 3 h, and MCP-1 and IL-8 protein in the culture media reached peak levels at 24 h. Therefore, the inhibitory effects of berberine on the MCP-1 and IL-8 mRNA were determined after stimulation of LPS at 3 h, and on the MCP-1 and IL-8 protein were determined after stimulation of LPS at 24 h.

In the present study, LPS dose-dependently stimulated MCP-1 and IL-8 expression in ARPE-19 cells. High levels of the MCP-1 and IL-8 in the aqueous humor and vitreous were involved in part in the pathogenesis of intraocular disorders such as age-related macular degeneration, proliferative vitreoretinal diseases, and diabetic retinopathy [18–25]. Therefore, the treatment of these eye diseases requires the suppression of MCP-1 and IL-8 expression.

In China, berberine (5–20 mg/kg per day) has long been used in the treatment of diarrhea and gastrointestinal disorders [27, 28]. In Japan, berberine alone is not used clinically. Mixed extracts from multiple herbs, which contain several alkaloids, are administered orally. For example, Oren-gedoku-to (Huang-Lian-Jie-Du-Tang in Chinese), which contains extracts from Scutellariae sp. radix, Coptidis sp. rhizoma, Gardeniae sp. fructus, and Phellodendri sp. cortex, is prescribed for treatment of gastritis. Oren-gedoku-to (1.5 g) usually contains berberine (240 mg). Kong et al. [42] reported that oral administration of berberine (0.5 g twice per day) for 3 months lowers serum low-density lipoprotein cholesterol in hypercholesterolemic people. The alkaloid also reported has the other pharmacological actions, including glucose-lowering potential [44–47].

We previously reported that berberine inhibited the in vivo expression of MCP-1 and IL-8 induced by LPS, decreasing the inflammatory cell infiltration and the levels of protein and cells in the aqueous humor in rat [36]. In the present study, we found that the alkaloid significantly inhibited the in vitro expression of MCP-1 and IL-8 induced by LPS in a human retinal pigment epithelial cell line. Therefore, an effect of berberine in the chemokine-mediated disorders may be expected.
